# Varicella zoster viral infection complicating into necrotizing fasciitis: A case report

**DOI:** 10.1002/ccr3.6408

**Published:** 2022-10-03

**Authors:** Nissar Shaikh, Umm‐e‐ Amara, Mogahed I. Hussein, Sahar Mahadik, Abdalaziz I. Elhussain, Muna Al Maslamani, Abdulqadir J. Nashwan

**Affiliations:** ^1^ Surgical Intensive Care Department Hamad General Hospital (HGH), Hamad Medical Corporation (HMC) Doha Qatar; ^2^ Apollo Institute of Medical Science and Research Hyderabad India; ^3^ Medical Education Department Hamad Medical Corporation (HMC) Doha Qatar; ^4^ Communicable Diseases Center (CDC) Hamad Medical Corporation (HMC) Doha Qatar; ^5^ Nursing Department, Hazm Mebaireek General Hospital (HMGH) Hamad Medical Corporation (HMC) Doha Qatar

**Keywords:** antibiotics, chicken pox, debridement, necrotizing fasciitis, shock, varicella zoster

## Abstract

Necrotizing fasciitis is a rare complication of varicella‐zoster viral infection in adults, occurring due to a secondary bacterial infection. A 35‐year‐old female healthy patient had post‐varicella zoster infection with NSAID use as a possible risk factor. She was diagnosed early by clinical and laboratory parameters.

## Graphical abstract

Chickenpox can cause life‐threatening infections, including meningitis and soft tissue infection. NSAIDs increase the risk of soft tissue infection. Early diagnosis and aggressive resuscitation with supportive organ care and antibiotic therapy are key to survival.

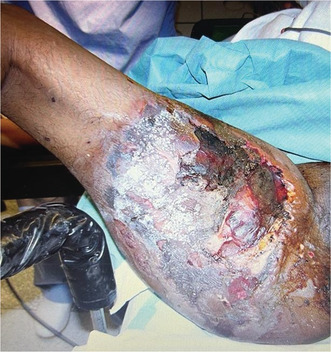

## 1 INTRODUCTION

Varicella zoster virus infection causes chickenpox in pediatric and adult immunocompetent patients. Around 47% of chicken pox infection occurs in the adult population, with male predominance.^1^ Chickenpox is usually a milder disease but can lead to serious neurological and respiratory complications and soft tissue infection.^2^ Less than 1% of children and 1.3% of adult patients with chicken pox will be complicated into necrotizing fasciitis (NF).^3^ Necrotizing fasciitis is a rare but potentially fatal skin and soft tissue infection and a surgical and medical emergency.^4^ We report a case of post chickenpox NF in an immunocompetent female adult patient.

## 2 CASE PRESENTATION

**FIGURE 1:**
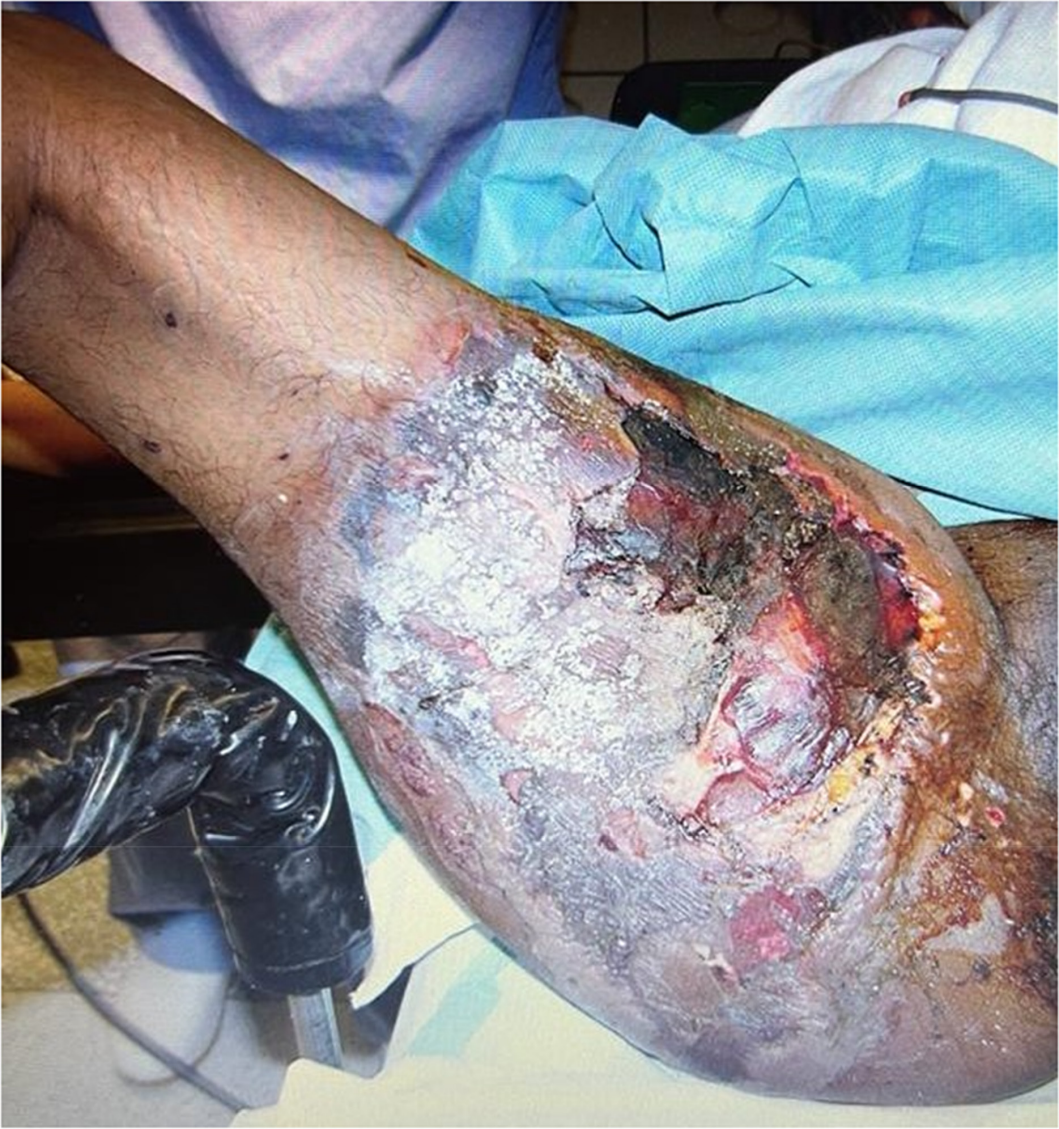
Thigh showing black colored skin lesion

**FIGURE 2:**
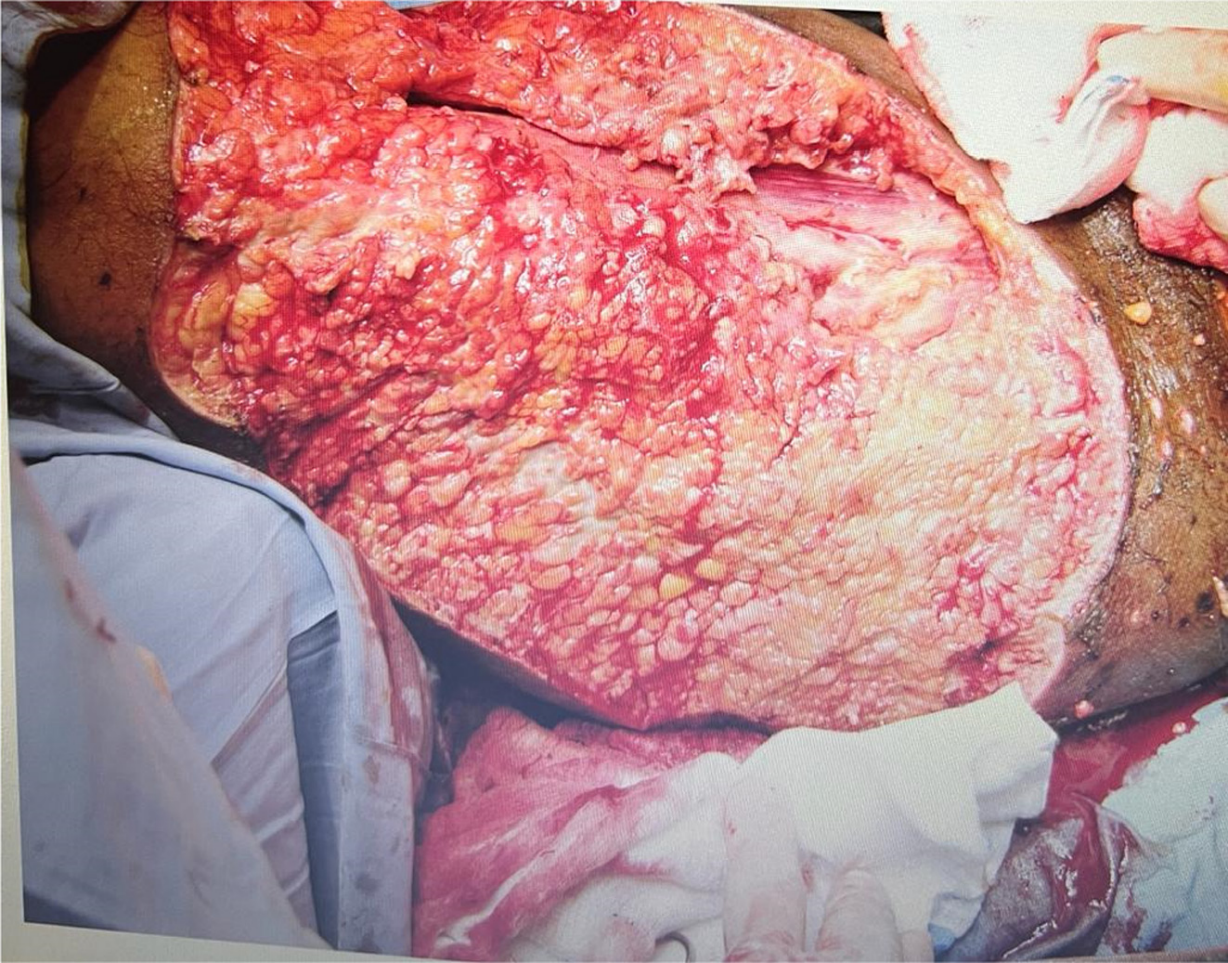
Post debridement of lesion

A 35‐year‐old female presented to the emergency department with severe pain and yellowish discharge in the left thigh for 2 days. She had a history of chickenpox for 2 weeks, diagnosed by typical skin rash, itchy, and progressively started from the abdomenand spread all over body associated with high‐grade fever and myalgia. Thigh rashes became painful, and she was taking regular ibuprofen, nonsteroidal anti‐inflammatory drug (NSAID), for a week. She was awake, dehydrated, tachycardiac (110–120 beats/min), tachypneic (24–29 breath/min), and febrile (39°C), with borderline blood pressure (90/50 mm Hg). On local examination, there were blackish lesions involving the posterior aspect of the left thigh, (Figure 1) extending to the perineum, vulva, and buttocks, with multiple blisters and yellowish discharge. Patient had severe tenderness in left thigh. Her laboratory workup showed leucocytosis (19 × 10^3^/μl), hyperglycemia (RBS 16.2 mmol/L), impaired renal function (BUN 14.5 mg/dl and serum creatinine 124 μmol/L), and anemia (Hb 7.2 g/dl) with high C‐reactive protein (324 mg/L). She was diagnosed as a case of NF by severe pain in thigh, high index of suspicion and use of abnormal basic laboratory parameters, LRINF score^5^ of 10 (Table 1), started on Tazocin® (piperacillin + tazobactam), and continued resuscitation with fluids and packed red blood cell transfusion (pRBCs). She was immediately taken for debridement of the thigh, necrotic tissues, and blackish skin lesions (Figure 2). Postoperatively, she was transferred to the surgical intensive care unit (SICU) in an intubated and ventilated condition. In the SICU, resuscitative measures were continued; she was in septic shock and required noradrenaline to maintain the hemodynamics. Clindamycin was added, and dalteparin was started for deep venous thrombosis prophylaxis. Tissue biopsy confirmed the diagnosis of NF. On day 2, she underwent re‐debridement and continued with resuscitation and supportive care. Tissue culture showed growth of *Streptococcus pyrogens* and *Pseudomonas aeruginosa*, both were sensitive to Tazocin®. By day 4, she was on enteral feeds, off vasopressors, and her trachea was extubated by day 5. The patient remained stable, all invasive lines were removed, and an oral diet was initiated. She was transferred to the surgical ward on day 7 and from there discharged home to be followed in outpatient clinics.

**TABLE 1:** Patient LRINF (laboratory risk indicators for necrotizing fasciitis) score

Patient LRINF score	
Serum sodium (serum Na)	132 mmol/L
Random blood sugar (RBS)	16.3 mmol/L
Hemoglobin (Hb)	7.2 g/dl
Leucocytosis (WBC)	19 × 10^3^/μl
C‐reactive proteins (CRP)	324 mg/L
Serum creatinine	124 μmol/L

## 3 DISCUSSION

Necrotizing fasciitis is a known rare complication of varicella zoster viral infection in adults, with an increased rate of complications compared to the pediatric age group.^1^ The central nervous system and soft tissue infections are the most frequent complications of chickenpox infection.^1^, ^2^, ^3^ In a recent study, 1.3% of chickenpox infections were complicated into NF.^6^ NF is a rapidly progressing infection of the fascial layer with delayed skin, subcutaneous, and muscle involvement with systemic toxicity.^4^ NF is classified into four classes depending on the microbiological etiology: class1 is polybacterial, class 2 is monobacterial Gram‐positive bacteria, class 3 is Gram‐negative marine monobacteria, and class 4 is fungal NF.^7^ In our patient, it was polybacterial, type1 NF. There are various risk factors for the occurrence of NF reported in the literature.^4^ Zerr et al.^8^ reported that the use of ibuprofen (NSAID) in children with varicella zoster infection had more NF, and our patients also were on regular ibuprofen before developing the NF. Although our patient was immunocompetent without any comorbidities, the regular use of ibuprofen may have increased the risk of NF, as with the use of NSAID, which impedes its timely recognition and management and accelerate the course of infection. Bryant et al.^9^ summarizes clinical and basic science evidence linking trauma and NSAID use to initiation and progression of severe GAS soft tissue infection.

Early diagnosis is key for better management of NF.^1^, ^3^ The most important finding in the patient's history and examination is severe pain, which will be much more intense and disproportionate to the local dermatological manifestations; this should raise a high index of suspicion for NF. The tissue biopsy is the gold standard for the diagnosis of NF. The LRINF score which is composed from the basic blood investigation (which are routinely done in the emergency department) helps in earlier diagnosis by differentiating NF from cellulitis.^1^, ^3^ Bechar et al.^10^ concluded their review by LRINEC significantly useful in the diagnosis of NF. More recently, Kazi et al. summarized their study by LRINEC score, which is reliable and easy‐to‐diagnose tool and the histopathology remains the gold standard for the diagnosis of NF. There was a statistically significant association between LRINF score and histopathology, and LRINEC was independently better than the bed side finger test in the diagnosis of NF.^11^

Can et al.^12^ reported a case of herpes zoster infection (diagnosed by typical distribution of skin rash) complicated by NF, difficult to diagnose NF, and diagnosed early and treated properly by LRINEC score. In our patient also, we suspected NF severe pain, blackish skin lesion, and diagnosed early by LRINEC score and immediately taken for debridement. Later, the tissue biopsy/histopathology confirmed the diagnosis of NF.

The management of NF is essentially medical as well as surgical. Medical management includes early antibiotic administration and organ supportive therapy, whereas surgical management is earlier bold debridement of the necrotic tissues.^4^, ^13^

## 4 CONCLUSION

Varicella zoster in healthy females can complicate into the NF, if they are on regular NSAID.

Necrotizing fasciitis can be diagnosed early with high index of suspicion, with severe pain disproportionate to the local skin manifestation and the use of LRINEC score. Early diagnosis in combination with surgical and medical therapy is key for better outcome in NF.

## AUTHOR CONTRIBUTIONS

Data collection and literature search: NSH. Manuscript preparation (draft and final editing): UA, MIH, SM, AIE, MAM, AJN. All authors read and approved the final manuscript.

## FUNDING INFORMATION

This study was not funded.

## CONFLICT OF INTEREST

The authors declare that they have no competing interests.

## ETHICAL APPROVAL

The article describes a case report. Therefore, no additional permission from our Ethics Committee was required (MRC‐04‐20‐1018).

## CONSENT

Written informed consent was obtained from the patients to publish this report in accordance with the journal's patient consent policy.

## Data Availability

All data generated or analyzed during this study are included in this published article.
